# Sperm is epigenetically programmed to regulate gene transcription in embryos

**DOI:** 10.1101/gr.201541.115

**Published:** 2016-08

**Authors:** Marta Teperek, Angela Simeone, Vincent Gaggioli, Kei Miyamoto, George E. Allen, Serap Erkek, Taejoon Kwon, Edward M. Marcotte, Philip Zegerman, Charles R. Bradshaw, Antoine H.F.M. Peters, John B. Gurdon, Jerome Jullien

**Affiliations:** 1 Wellcome Trust/Cancer Research UK Gurdon Institute, University of Cambridge, Cambridge, CB2 1QN, United Kingdom;; 2 Department of Zoology, University of Cambridge, Cambridge, CB2 3EJ, United Kingdom;; 3 Friedrich Miescher Institute for Biomedical Research, 4058 Basel, Switzerland;; 4 Faculty of Sciences, University of Basel, 4001 Basel, Switzerland;; 5 Department of Molecular Bioscience, Center for Systems and Synthetic Biology, Institute for Cellular and Molecular Biology, The University of Texas at Austin, Austin, Texas 78712, USA

## Abstract

For a long time, it has been assumed that the only role of sperm at fertilization is to introduce the male genome into the egg. Recently, ideas have emerged that the epigenetic state of the sperm nucleus could influence transcription in the embryo. However, conflicting reports have challenged the existence of epigenetic marks on sperm genes, and there are no functional tests supporting the role of sperm epigenetic marking on embryonic gene expression. Here, we show that sperm is epigenetically programmed to regulate embryonic gene expression. By comparing the development of sperm- and spermatid-derived frog embryos, we show that the programming of sperm for successful development relates to its ability to regulate transcription of a set of developmentally important genes. During spermatid maturation into sperm, these genes lose H3K4me2/3 and retain H3K27me3 marks. Experimental removal of these epigenetic marks at fertilization de-regulates gene expression in the resulting embryos in a paternal chromatin-dependent manner. This demonstrates that epigenetic instructions delivered by the sperm at fertilization are required for correct regulation of gene expression in the future embryos. The epigenetic mechanisms of developmental programming revealed here are likely to relate to the mechanisms involved in transgenerational transmission of acquired traits. Understanding how parental experience can influence development of the progeny has broad potential for improving human health.

Embryos obtained by fertilization develop to adulthood more frequently than those obtained by other methods, such as nuclear transfer (Gurdon et al. 1958; Kimura and Yanagimachi 1995), suggesting that sperm is specially programmed to support normal embryonic development. Several hypotheses were proposed to explain the nature of this programming, including the idea that sperm is programmed for efficient replication after fertilization (Lemaitre et al. 2005) or for supporting proper embryonic transcription (Suzuki et al. 2007; Hammoud et al. 2009; Zheng et al. 2012). The latter hypothesis was proposed following the observation that promoters of developmentally important genes escape global replacement of histones by protamines in mature sperm. In fact, these promoters retain post-translationally modified histones, suggesting that epigenetic marks on sperm chromatin may be transmitted to the embryo at fertilization and could subsequently pattern transcription of embryonic genes (Suzuki et al. 2007; Hammoud et al. 2009; Brykczynska et al. 2010; Wu et al. 2011; Paradowska et al. 2012; Zheng et al. 2012; Ihara et al. 2014; Siklenka et al. 2015). However, the validity of this hypothesis was recently questioned (Carone et al. 2014; Samans et al. 2014).

In this work, we compared the developmental potential of sperm to that of spermatids in order to understand the nature of sperm programming for development in *Xenopus laevis*. The use of spermatids, immediate precursors of sperm, suits such comparisons because (1) spermatids have completed meiosis and have the same DNA content as sperm, and (2) spermatid chromatin structure resembles that of somatic cells (Gaucher et al. 2010). Furthermore, in the mouse, embryos derived from injection of spermatids into unfertilized oocytes develop to adulthood less frequently than embryos derived from injection of sperm, suggesting that developmentally important information is acquired during spermatid to sperm maturation (Kimura and Yanagimachi 1995; Kishigami et al. 2004). Here, we demonstrate that sperm is epigenetically programmed to regulate transcription of several developmentally important embryonic genes.

## Results

### Sperm-derived embryos develop better than spermatid-derived embryos

**Figure 1.**
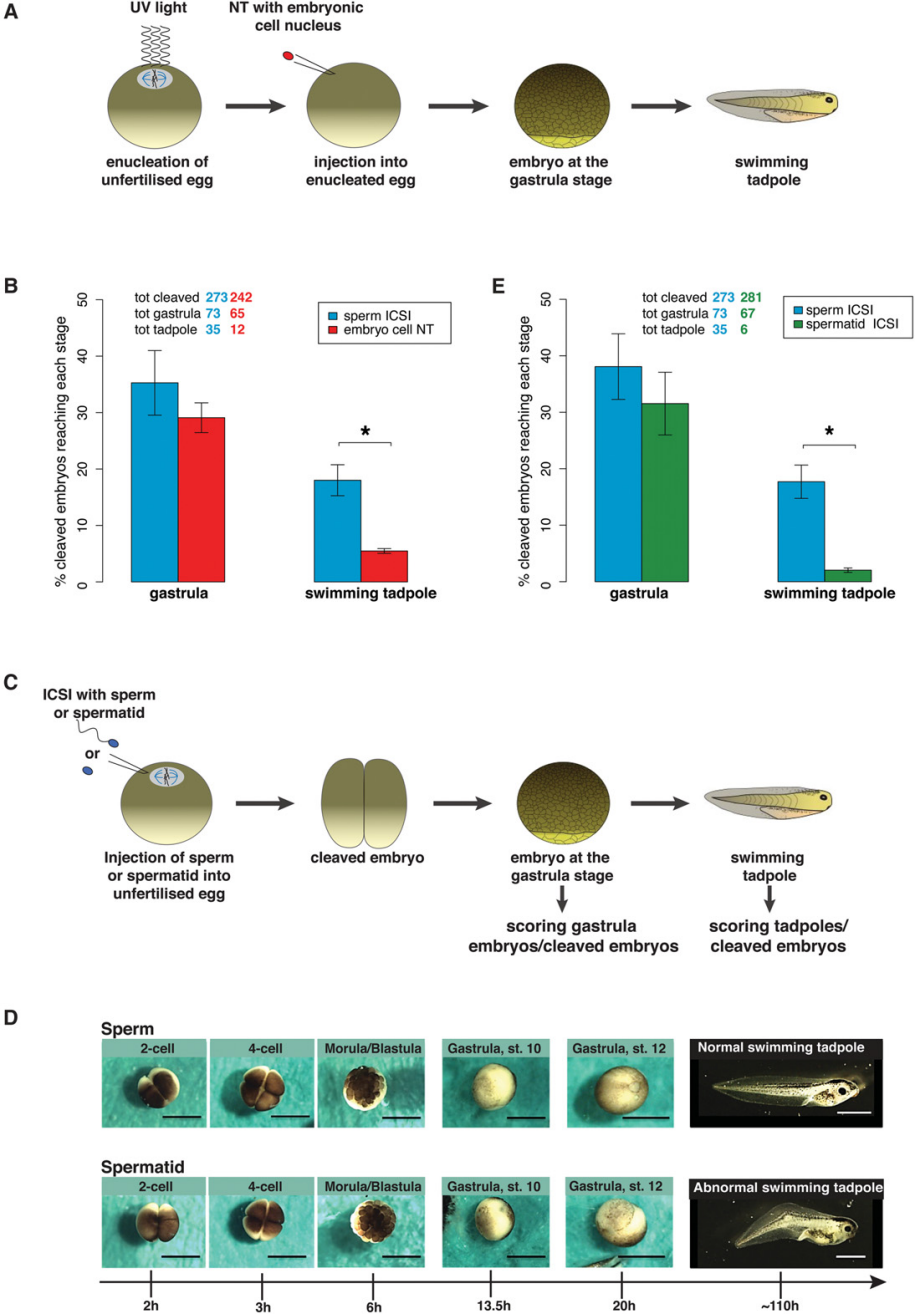
*Xenopus* sperm is better at supporting development than a spermatid or a somatic cell. (*A*) Experimental design for the generation of cloned embryos. The somatic nucleus of a gastrula cell is transplanted to a UV-enucleated egg. The resulting embryos are scored at the gastrulation and tadpole stage. (*B*) Scoring of embryos as % of gastrulae and as % of swimming tadpoles to the total number of cleaved embryos. Average of *n* = 6 independent experiments (sperm ICSI), and *n* = 3 independent experiments (embryo cell NT). The total number of embryos analyzed is shown *above* the graph. Error bars: SEM. (*) *P*-value < 0.05 (χ^2^ test). (*C*) Experimental design for the generation of sperm- and spermatid-derived embryos. Permeabilized sperm or spermatids are injected to the cytoplasm (ICSI) of an unfertilized egg. The resulting embryos are scored at the gastrulation and tadpole stage. (*D*) Representative images of sperm- and spermatid-embryos. Scale bars = 1 mm. (*E*) Scoring of embryos as % of gastrula and as % of swimming tadpoles to the total number of cleaved embryos (average of *n* = 6 independent experiments). The total number of embryos analyzed is shown *above* the graph. Error bars: SEM. (*) *P*-value < 0.05 (χ^2^ test).

We first compared the development of embryos obtained by transplanting somatic cell nuclei (SCNT) with the development of sperm-derived embryos. To minimize the experimental difference in the way the embryos were generated, both types of embryos were generated by nuclear injection: cloned embryos were obtained by transplanting nuclei of embryonic cells to enucleated eggs (Fig. 1A), and sperm-derived embryos were produced by intra-cytoplasmic injection of sperm (ICSI) to eggs (Fig. 1C). We observed that cloned embryos developed less efficiently to the swimming tadpole stage than sperm-derived embryos (Fig. 1B). This illustrates the better developmental potential of sperm over that of a somatic cell. In this experimental set-up, however, the way the embryos are generated is quite different: the maternal genome is present in sperm-derived embryos, whereas it has been removed in the SCNT embryos. In order to better assess the developmental potential of sperm, we aimed to compare embryos produced in the same way. For that purpose, we generated embryos by injecting permeabilized purified sperm or spermatids (Supplemental Fig. S1) to the cytoplasm of unfertilized eggs (ICSI) (Fig. 1C). In that way, both types of embryos are obtained in the same manner, and their development can be compared. The two types of embryos reached the gastrula stage with a similar frequency. However, sperm-derived embryos developed significantly better to the swimming tadpole stage than spermatid-derived ones (*P*-value < 0.05) (Fig. 1D,E). This is in agreement with observations made previously in mouse (Kimura and Yanagimachi 1995; Kishigami et al. 2004). Spermatid-derived embryos and cloned embryos exhibit a similar reduction in developmental potential when compared to sperm-derived embryos (Fig. 1B–E), suggesting that the spermatid is as severely impaired to support development as a somatic cell.

In conclusion, embryos can be generated in the same way from sperm or spermatids, and spermatids show reduced developmental potential compared to sperm. Therefore, the comparison of sperm and spermatids was subsequently used to investigate why sperm support better development than other cell types.

### Spermatids replicate their DNA as efficiently as sperm

**Figure 2.**
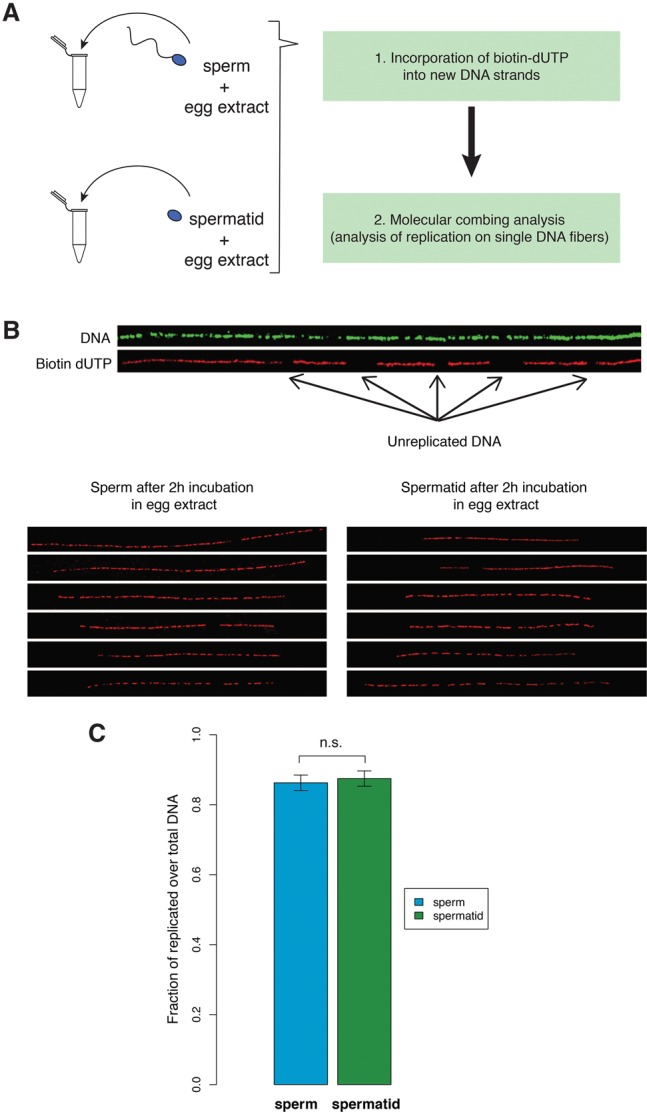
Spermatids are as good as sperm at DNA replication. (*A*) Sperm and spermatids are separately incubated with egg extracts supplemented with biotin-dUTP. Subsequently, DNA fibers are isolated and subjected to molecular combing, which reveals replication on single DNA fibers. (*B*) Examples of DNA fibers after immunostaining procedure. Antibody staining against DNA reveals the total length of the fiber (green) and antibody staining against biotin reveals the replicated DNA (red). The *bottom* panels show representative examples of replication staining from sperm and from spermatids incubated in egg extracts. (*C*) Replication extent measured as the proportion of DNA that incorporated biotin-dUTP to the total fiber length. Results are from at least 125 independent DNA fibers (22,000 kb of DNA for each sample). Error bars: SEM. Samples were not significantly different (*P*-value = 0.41, KS-test).

Since it has been shown that sperm, as opposed to other cells, replicate DNA more efficiently (Lemaitre et al. 2005), we hypothesized that the poor embryonic development of spermatid-derived embryos is due to inefficient DNA replication. Egg extracts from *Xenopus* have been widely used to investigate mechanisms of replication (Hutchison et al. 1989; Lemaitre et al. 2001). By incubating nuclei in egg extracts, one can mimic replication events that occur prior to the first embryonic cell division. We measured DNA replication efficiency in sperm and spermatids incubated in egg extracts (Hutchison et al. 1989) by molecular combing analysis (Gaggioli et al. 2013). In this assay, the egg extract is supplemented with modified nucleotide precursors that will be incorporated into replicating DNA (Fig. 2A). Following replication, DNA is stretched on a slide, and both the total (green) and replicated (red) DNA fibers are fluorescently labeled (Fig. 2B). By measuring the extent of replication on several hundreds of DNA fibers, we observed equally efficient DNA replication in both sperm and spermatids (Fig. 2C). We concluded from this analysis that the nature of sperm programming is not related to replication. We then tested whether spermatid-derived embryos are capable of correctly initiating embryonic transcription.

### Haploid sperm-derived embryos develop better than haploid spermatid-derived embryos

**Figure 3.**
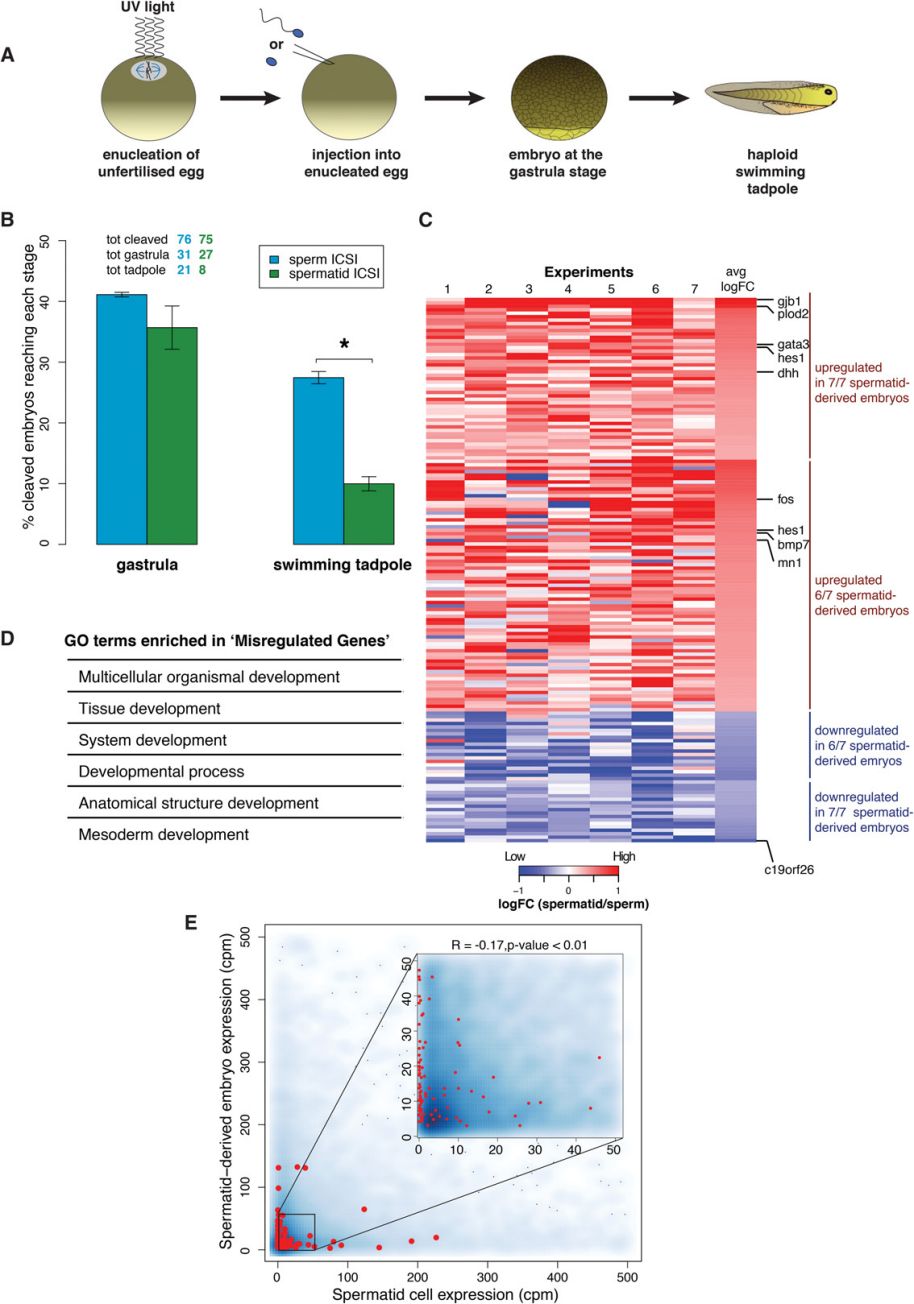
Transcription of developmentally important genes is misregulated in spermatid-derived embryos compared to sperm-derived embryos. (*A*) Schematic representation of paternally derived haploid embryos generated by UV enucleation of eggs followed by intra-cytoplasmic sperm injection (ICSI). (*B*) Developmental advantage of sperm over spermatid is maintained in haploid embryos. Embryos were scored as the % of embryos reaching a gastrula stage and a swimming tadpole stage to the total number of cleaved embryos (average of *n* = 3 independent experiments). Numbers of embryos analyzed are indicated *above* the bars. Error bars: SEM. (*) *P*-value < 0.05 (χ^2^ test). (*C*) Genes important for development are misregulated (mostly up-regulated) in spermatid-derived embryos. Heat map representing log fold-change in expression levels of the 100 genes (rows) misregulated in spermatid versus sperm gastrula embryos (FDR < 0.05; red: up-regulated; blue: down-regulated in spermatid) across seven independent experiments (columns). (*D*) Developmentally important gene ontology terms enriched in the list of misregulated genes (*P*-value < 0.05). (*E*) Up-regulation of genes in spermatid-derived embryos does not correlate with their transcription in spermatid. Density scatter plot showing gene expression in spermatid-derived embryos versus that in spermatids. No correlation is observed between the two parameters for all genes (*r* = 0.06) as well as for the misregulated genes (red dots, *r* = −0.17).

To rigorously assess the developmental potential and transcriptional ability of sperm and spermatids, and to eliminate the risk of any potential interference from the maternal nucleus, we used haploid, paternally derived embryos generated by injection of permeabilized sperm or spermatids into enucleated eggs (Fig. 3A; Supplemental Fig. S2; Narbonne et al. 2011). We first confirmed that haploid, sperm-derived embryos developed significantly better to the swimming tadpole stage than haploid, spermatid-derived embryos (*P*-value < 0.05) (Fig. 3B), recapitulating the results from diploid embryos (Fig. 1E). Previous mammalian experiments comparing developmental potential of sperm and spermatids used diploid biparental embryos (Kimura and Yanagimachi 1995; Kishigami et al. 2004). Our results with paternal haploid embryos directly demonstrate that the sperm nucleus supports better development than the spermatid nucleus, with or without the maternal genome. Furthermore, this experimental set-up allows a specific assessment of transcription originating exclusively from sperm- or spermatid-derived chromatin at the time of embryonic gene activation.

### Developmentally important genes are misregulated in spermatid-derived embryos

Since experiments in the mouse suggested that sperm might be better than other cell types at supporting mRNA transcription (Ziyyat and Lefevre 2001; Vassena et al. 2007; Ihara et al. 2014), we tested this hypothesis using haploid, sperm- and spermatid-derived embryos. Embryos were rigorously staged (see Supplemental Experimental Procedures) and collected at gastrulation, before the onset of developmental defects. Gene expression was then analyzed by RNA-seq, using a set of 34,373 transcripts (provided as Supplemental Gene Annotation). Out of 18,340 expressed genes, 255 were differentially expressed in spermatid-derived embryos compared to sperm-derived embryos (FDR < 0.05) (Supplemental Table S1). One hundred of these transcripts showed consistent changes in at least six out of seven experiments, and we will refer to them as “misregulated” (Fig. 3C; Supplemental Table S1). The majority (82/100) were up-regulated in spermatid-derived embryos, and they include transcriptional regulators (e.g., *gata2*, *gata3*, *hes1*, and *fos*) as well as morphogens (e.g., *bmp2*, *bmp7*, or *dhh*) essential for embryonic development. Accordingly, gene enrichment analysis revealed that several development-related terms were significantly enriched in the list of misregulated genes (*P*-value < 0.05) (Fig. 3D).

The fact that most of the misregulated genes were up-regulated in spermatid-derived embryos raised the possibility that these genes were actively transcribed in spermatids and continued to be transcribed in embryos. Indeed, the spermatid, as opposed to the sperm, is a transcriptionally active cell, and this difference might explain why genes are up-regulated in embryos originating from spermatids rather than sperm. To test this possibility, we compared the expression level of genes in spermatids and in spermatid-derived embryos. We did not observe any correlation between the expression of misregulated genes in spermatid-derived embryos and their expression in spermatids (*r* = −0.17, *P*-value < 0.05) (Fig. 3E). This suggests that the up-regulation of these genes in spermatid-derived embryos is not the result of transcript carry-over (or ongoing transcription) from spermatid chromatin. Because permeabilized spermatids used to generate embryos are likely to contain additional RNAs, we performed an additional control for the potential effect of spermatid-derived RNAs on embryonic development. We purified total RNA from testis and injected a quantity corresponding to the amount found in one spermatid (50 pg) to embryos generated with sperm. TRIzol was used to isolate RNA as it allows the recovery of RNA in a broad range of sizes (El-Khoury et al. 2016). All embryos generated in that way developed normally, indicating that testicular RNAs are not detrimental to embryonic development (Supplemental Fig. S3). Lastly, we have verified that the synthesis of rRNAs is not affected in spermatid-derived embryos. Indeed, in mouse, defect in rRNA synthesis has been proposed to explain the developmental defect of nuclear transfer embryos (Suzuki et al. 2007; Zheng et al. 2012) and could explain the difference in the bulk of RNA synthesis observed between sperm- and spermatid-derived embryos (Bui et al. 2011). We observed that newly synthesized 18S and 28S rRNAs are equally well produced in sperm- and spermatid-derived embryos (Supplemental Fig. S4).

We conclude that the developmental failure of spermatid-derived embryos is not associated with carried over spermatid RNAs or with defects in rRNA expression. Rather, we observe a correlation between developmental defects and misexpression of a set of developmentally important genes in spermatid-derived embryos. We hypothesized that differences in gene expression between sperm- and spermatid-derived embryos might result from epigenetic differences of sperm/spermatid chromatin.

### Epigenetic differences between sperm and spermatid chromatin

**Figure 4.**
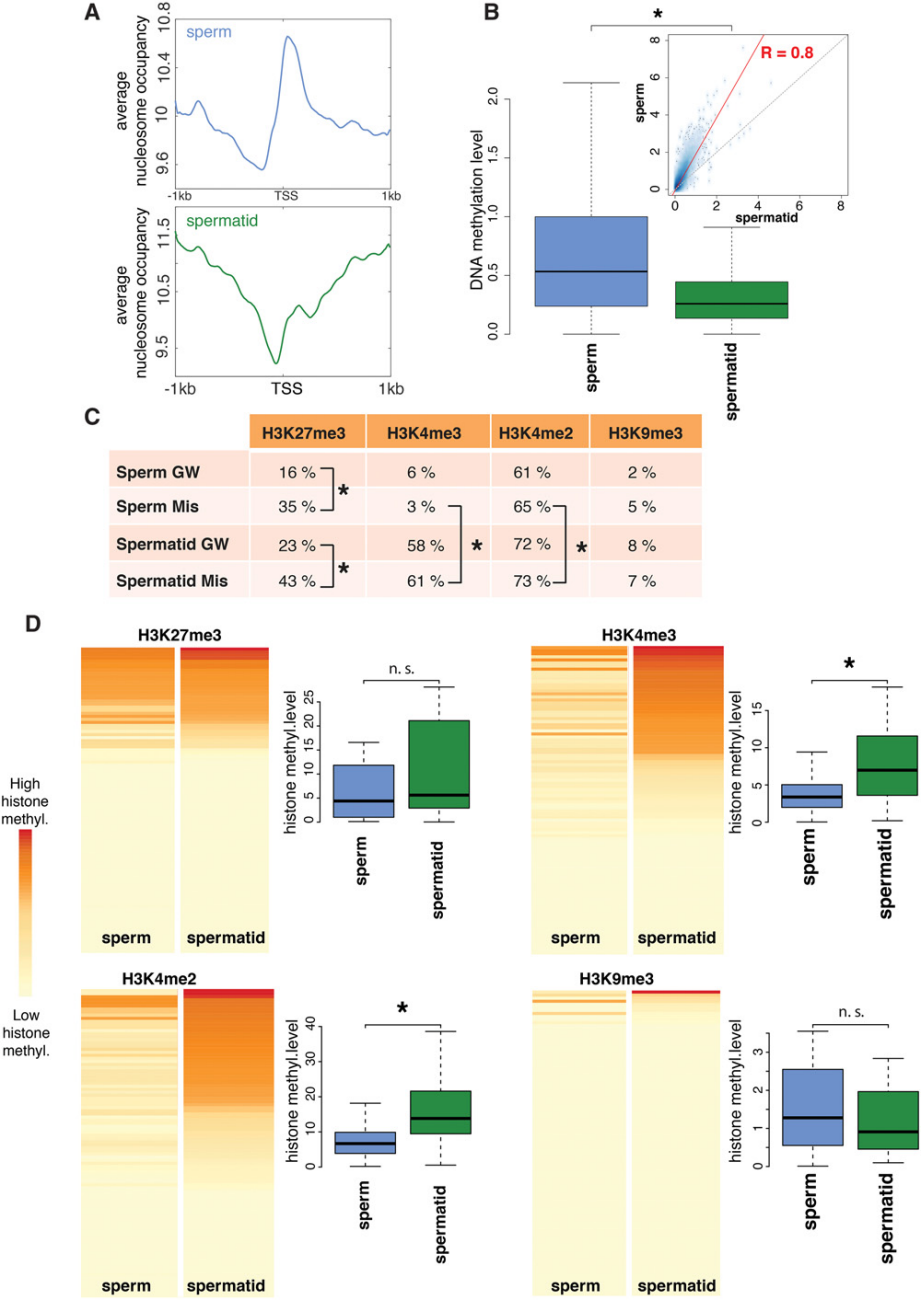
Genes that are misregulated in spermatid-derived embryos have different epigenetic features in sperm and spermatid. (*A*) Genome-wide average nucleosome occupancy at the TSS of sperm (blue) and spermatid (green) genes. (*B*) Boxplots showing genome-wide DNA methylation levels at the TSS ± 1 kb of sperm (blue) and spermatid (green) genes. *Inset* shows correlation between the DNA methylation levels of sperm and spermatid (*R* = 0.8, *P*-value < 0.05); red line: regression; dotted line: diagonal. (*C*) Percentage of genes harboring H3K27me3, H3K4me3, H3K4me2, or H3K9me3 peaks genome-wide (GW) and at misregulated genes (Mis). (*) *P*-value < 0.05 (χ^2^ test). (*D*) Heat maps representing H3K27me3, H3K4me3, H3K4me2, and H3K9me3 overall levels (see Supplemental Material and Supplemental Fig. S8) at misregulated genes in sperm (first column) and spermatid (second column). Each map is sorted according to the signal in spermatid. Boxplots show the distribution of methylation levels across misregulated genes. (*) *P*-value < 0.05 (KS-test) (Supplemental Table S7).

To investigate potential links between the epigenetic marking of paternal chromatin and embryonic gene expression, we carried out epigenetic profiling of mononucleosomal chromatin from sperm and spermatid using an extensive MNase digestion protocol applied by others to probe for histones stably associated with chromatin in mature sperm in mouse and human (Supplemental Tables S2, S3; Supplemental Fig. S5; Hammoud et al. 2009; Brykczynska et al. 2010). In *Xenopus*, the transition from spermatid to sperm is characterized by histone H3 and H4 retention and partial loss of H2A and H2B (Risley and Eckhardt 1981). We first compared nucleosome occupancy profiles in sperm and spermatids. Similarly to what was observed in other vertebrates (Hammoud et al. 2009; Brykczynska et al. 2010), we observed higher nucleosomal occupancy (MNase-seq) (Fig. 4A) around TSSs (transcriptional start sites) in sperm when compared to spermatids. In this context, the positioned nucleosomes show a similar distribution in sperm and spermatids (Supplemental Fig. S6). Secondly, we analyzed DNA methylation profiles in sperm and spermatids by MBD-seq. DNA methylation was higher around sperm TSSs than spermatid TSSs (Fig. 4B). These differences were observed at the genome-wide level between sperm and spermatids, as well as on the set of misregulated genes (Supplemental Figs. S6, S7). A lower level of nucleosome occupancy and DNA methylation in spermatids compared to sperm could therefore explain the up-regulation of genes in spermatid- compared to sperm-derived embryos.

To further characterize sperm and spermatid chromatin, we performed ChIP-seq analysis of histone marks associated with activation (H3K4me2, H3K4me3) or repression (H3K27me3, H3K9me3) of transcription. We looked for peaks (Fig. 4C; Supplemental Table S4) as well as for the overall methylation levels (Fig. 4D; Supplemental Fig. S8; Supplemental Table S5) around TSSs for each of these modifications. When compared to all genes, the set of misregulated genes was significantly enriched for H3K27me3 in both sperm and spermatids (Fig. 4C). However, since H3K27me3 was present in both sperm and spermatids, it suggests that this repressive mark alone cannot explain the difference in gene expression observed in sperm- and spermatid-derived embryos.

Interestingly, histone marks associated with active transcription (H3K4me2/3) showed an enrichment at promoters of misregulated genes in spermatids but not in sperm (Fig. 4C,D), providing a plausible explanation for the up-regulation of these genes in spermatid-derived embryos.

### Coexistence of H3K4me2/3 and H3K27me3 in spermatids correlates with embryonic gene up-regulation

**Figure 5.**
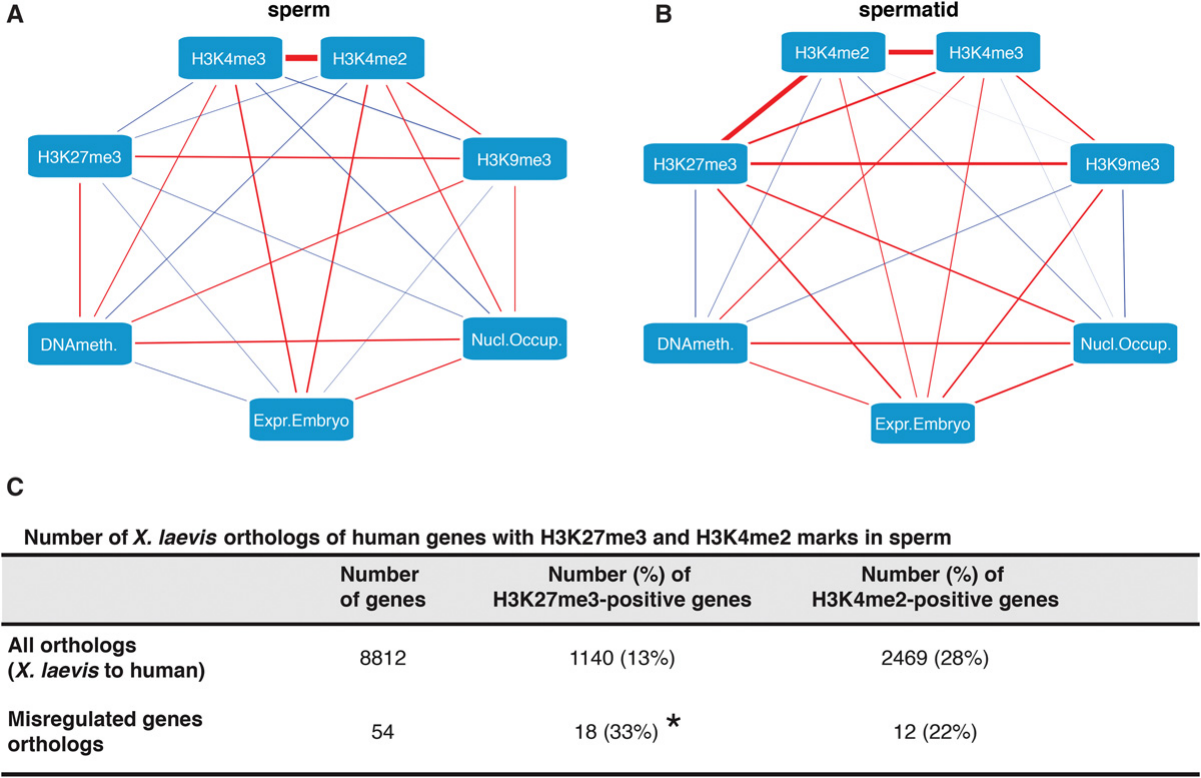
H3K27me3 target genes that lose H3K4me2/3 in sperm compared to spermatids are misregulated in spermatid-derived embryos. (*A*,*B*) Differential gene expression between sperm- and spermatid-derived embryos best correlates with differential H3K4me2/3 and H3K27me3 marking in sperm and spermatids. Partial correlation network between all tested epigenetic features of the paternal chromatin (*A*, sperm; *B*, spermatid) and gene expression in the corresponding embryos. Edges (lines) represent positive (red) or negative (blue) partial correlations. Edges thickness: strength of the partial correlations. (*C*) H3K4me2 and H3K27me3 marking on misregulated genes is conserved between *Xenopus* and human sperm. As compared to all orthologs, the misregulated orthologs are enriched for H3K27me3 marks over the genome-wide average in human sperm (χ^2^ test, [*] *P*-value < 0.05). No statistical enrichment for H3K4me2 on misregulated genes as compared to the genome-wide average is observed in human sperm.

The epigenetic features analyzed (histone marks, DNA methylation, and nucleosome occupancy) can individually provide a possible explanation for the observed differences in expression in sperm- and spermatid-derived embryos. However, complex interactions involving more than one epigenetic feature might better explain this differential embryonic gene expression. In order to identify such interactions, we have performed a partial correlation analysis. In this analysis, all the measured parameters are assessed simultaneously to produce maps indicating the way epigenetic features associate with each other and with gene expression. The aim of such analysis is to extract more general principles describing the paternal epigenetic program underlying gene expression and identify which epigenetic features are likely to have the strongest contribution to embryonic gene expression. We applied the partial correlation analysis to identify links between the measured epigenetic features in sperm and the expression of the misregulated genes in sperm-derived embryos (Fig. 5A), or between the measured epigenetic features in spermatid and their expression in spermatid-derived embryos (Fig. 5B). We observed that sperm H3K4me2/3 and embryonic gene expression were positively associated, whereas sperm H3K27me3, H3K9me3, and DNA methylation are negatively linked to embryonic gene expression (Fig. 5A). Interestingly, activating H3K4me2/3 and repressive H3K27me3 marks in spermatids were positively linked to gene expression in spermatid-derived embryos, and at the same time they were also strongly associated with each other (Fig. 5B). These associations were also observed when performing a similar analysis using an extended set of misregulated genes obtained by relaxing the selection parameters from FDR ≤ 0.05 (255 genes) to FDR ≤ 0.4 and |logFC| ≥ 0.2 (1116 genes). The use of this extended set increased the predictive power of the analysis and showed stronger links between the features tested within an overall similar network (Supplemental Fig. S9). Therefore, the difference between sperm- and spermatid-derived embryos is best explained by the fact that, in contrast to sperm, where H3K27me3 is overrepresented at genes differentially expressed in haploid embryos, in spermatids H3K27me3 coexists with H3K4me2/3 on these genes, thereby contributing to their up-regulation in spermatid-derived embryos.

We checked whether the observed distribution of H3K4me2/3 and H3K27me3 on the misregulated genes of *Xenopus* sperm was a conserved feature across species. For that purpose, we investigated how these histone marks were distributed in human sperm (Hammoud et al. 2009) on human orthologs of the *Xenopus* misregulated genes. We observed that, similarly to *Xenopus* sperm, human sperm showed an enrichment for H3K27me3 on misregulated genes (Fig. 5C). Additionally, misregulated genes did not exhibit any enrichment over the genome-wide distribution for H3K4me2 in both species. This indicates a conservation of sperm epigenetic features on these genes in the two species.

We next tested if paternally derived H3K4me2/3 and H3K27me3 were indeed involved in patterning embryonic gene expression.

### Paternal H3K4me2/3 and H3K27me3 influence embryonic gene expression

**Figure 6.**
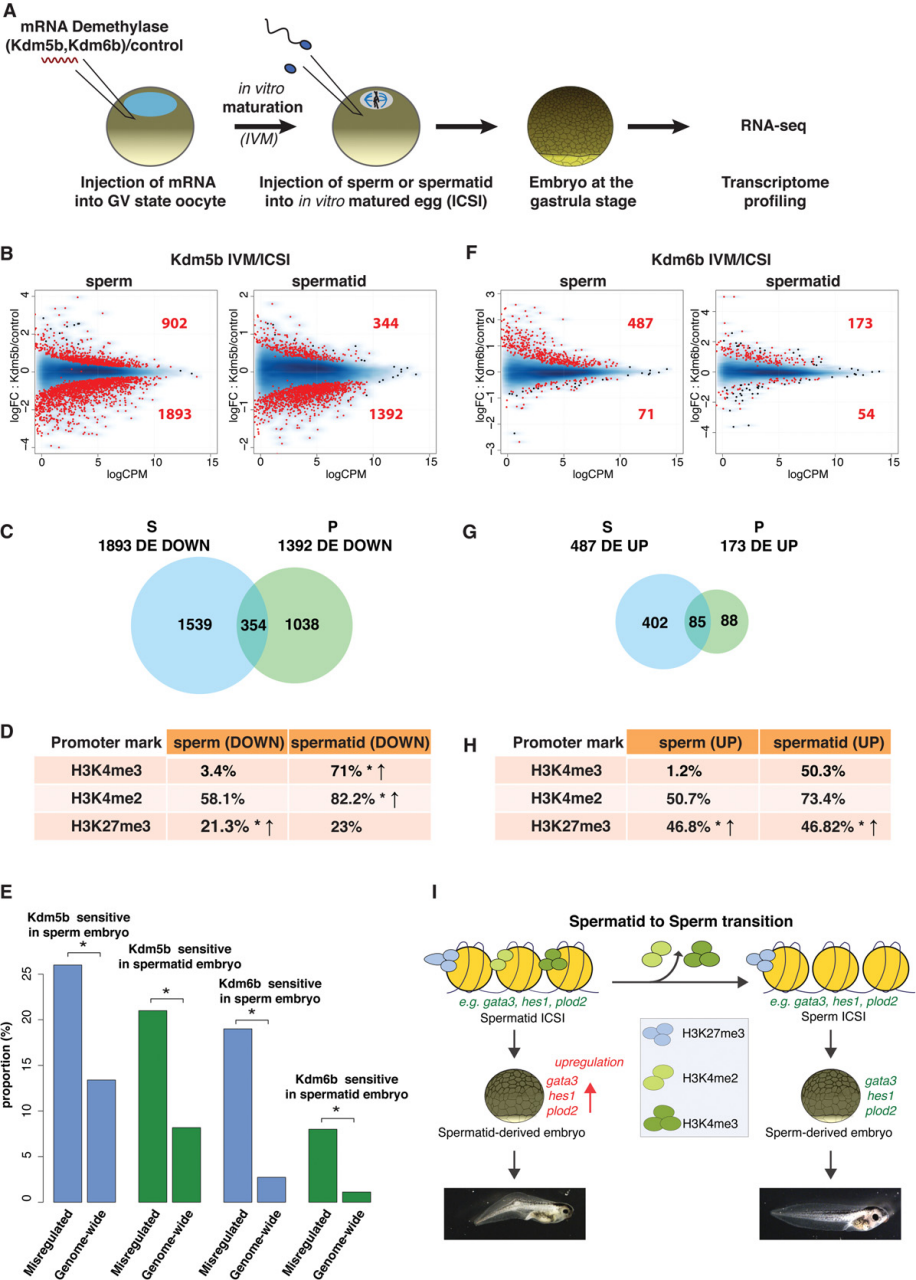
Paternal genome marking by H3K4me2/3 and H3K27me3 is required for gene expression in the embryos. (*A*) Histone demethylase expression assay. (*B*) MA plot showing log fold-change (logFC, *y*-axis) in gene expression between *Kdm5b* (H3K4me2/3 demethylase)- versus control mRNA-injected embryos, against log counts per million (logCPM, *x*-axis). Red dots: genes differentially expressed (FDR < 0.05); *N* = 4 independent experiments. (*C*) Venn diagram of down-regulated genes upon KDM5B expression in sperm- (blue) and spermatid-derived (green) embryos. (*D*) Percentages of genes down-regulated upon KDM5B expression in embryos that show H3K4me2/3 and H3K27me3 promoter peaks in the paternal cell. (*) *P*-value < 0.05 (χ^2^ test); ↑: over-represented when compared to genome-wide distribution. (*E*) Proportion of misregulated genes affected in each demethylase expression assay. (*) *P*-value < 0.05 (χ^2^ test). (*F*) Same as *B* for KDM6B (H3K27me3 demethylase) expression. (*G*) Same as *C* with genes up-regulated upon KDM6B expression. (*H*) Same as *D* for genes up-regulated upon KDM6B expression. (*I*) Model of epigenetic programming of sperm for the regulation of embryonic transcription.

To test the function of epigenetic marks from sperm or spermatid chromatin on the regulation of embryonic transcription, we experimentally removed these marks in embryos (Fig. 6A; Supplemental Fig. S10). mRNAs encoding histone demethylases or control mRNAs were first injected into immature oocytes. After allowing 24 h for the enzymes to be expressed, the oocytes were in vitro–matured into eggs (IVM) and injected with sperm or spermatids (ICSI). The resulting embryos were collected at the gastrulation stage for RNA-seq analysis. In this protocol, histones from both maternal and paternal chromatin are demethylated when the embryo is generated. By comparing embryos produced with different paternal chromatin (sperm or spermatid), we can evaluate the effect of paternal epigenetic mark removal on embryonic gene expression.

We first expressed the H3K4me2/3 demethylase, KDM5B. As expected, removal of the activating H3K4me2/3 marks led to gene down-regulation: 68% (1893 genes) and 80% (1392 genes) of all differentially expressed (FDR < 0.05) genes were down-regulated in sperm- and spermatid-derived embryos, respectively (Fig. 6B; Supplemental Table S6). Importantly, genes down-regulated in sperm-derived embryos showed only limited overlap with those down-regulated in spermatid-derived embryos (Fig. 6C). This indicates a paternal chromatin-dependent effect of H3K4me2/3 removal on embryonic gene expression. Additionally, among the genes affected by H3K4me2/3 removal in sperm- and spermatid-derived embryos, the misregulated genes are overrepresented, indicating that paternal H3K4me2/3 specifically regulates this set of genes (Fig. 6E). Interestingly, the genes down-regulated in spermatid-derived embryos are enriched for H3K4me2/3 in spermatids (Fig. 6D). These observations are in agreement with the hypothesis that the loss of H3K4me2/3 from H3K27me3 marked genes during the spermatid to sperm maturation is necessary for their proper expression in embryos.

To further validate this hypothesis, we tested the influence of paternal H3K27me3 by overexpressing the H3K27me3 demethylase KDM6B (Fig. 6A). In accordance with its repressive function, removal of H3K27me3 at fertilization led to up-regulation of genes at gastrulation in both sperm- and spermatid-derived embryos. Eighty-seven percent (487 genes) and 76% (173 genes) of differentially expressed genes (FDR < 0.05) were up-regulated in sperm- and spermatid-derived embryos, respectively (Fig. 6F; Supplemental Table S6). Again, there was only a partial overlap between genes affected by KDM6B in sperm- and spermatid-derived embryos (Fig. 6G), indicating that this effect is paternal chromatin-dependent. The affected genes were marked by H3K27me3 in the corresponding paternal cells (Fig. 6H). Additionally, upon H3K27me3 demethylation, about five times more genes were specifically up-regulated in sperm- than in spermatid-derived embryos (402 versus 88 genes). This suggests that the programming of genes for embryonic expression in the paternal chromatin relies on the establishment of an effective H3K27me3-mediated repression at the spermatid to sperm transition. Lastly, the misregulated genes are enriched among the genes affected by the H3K27me3 removal, indicating that paternal H3K27me3 specifically regulates this set of genes (Fig. 6E).

## Discussion

Previous work characterizing the epigenetic features of sperm in zebrafish, mouse, and human has revealed the presence of modified histones around genes involved in embryonic development (Hammoud et al. 2009; Brykczynska et al. 2010; Wu et al. 2011). In these species, the presence of activating (H3K4me3) and repressive (H3K27me3) histone marks in sperm correlated with gene expression in the early embryos (Hammoud et al. 2009; Brykczynska et al. 2010; Wu et al. 2011). In this work, we have used a comparison of sperm and its immediate precursor, the spermatid, to investigate the functional relationship between histone marks and gene expression.

Our analysis shows that, similar to what has been observed in mouse (Kimura and Yanagimachi 1995; Kishigami et al. 2004), spermatids are not as good at supporting development as sperm. Second, we tested several hypotheses proposed to explain the developmental advantage of sperm over spermatids. We have ruled out the hypothesis that spermatids are less efficient than sperm at supporting replication. Instead, we found evidence supporting the hypothesis that sperm is programmed to support proper embryonic expression of genes encoding important embryonic regulators (Fig. 3). Importantly, overexpression and knockdown studies of several of these genes have shown embryonic developmental defects reminiscent of what is observed in spermatid-derived embryos (*sfrp2* [Lee et al. 2006]; *tbx3* [Weidgang et al. 2013]; *foxa2* [Suri et al. 2004]; *otx2* [Yasuoka et al. 2014]). These observations suggest that misexpression of this set of genes is the cause of the developmental defect observed in spermatid-derived embryos. We also showed that the developmental advantage of sperm over spermatids is maintained in haploid, paternally derived embryos, indicating that the effect observed is independent of the presence of the maternal genome. To our knowledge, this is the first time that these two hypotheses, developmental advantage related to ability to support replication versus transcription, have been rigorously tested.

These analyses allowed us to conclude that sperm is not merely a carrier of DNA, but that it also contributes epigenetic information required for proper embryonic gene expression. We then focused our analysis on the sperm chromatin as it represents the most likely vector of such epigenetic information.

During spermiogenesis in *Xenopus laevis*, core histones H3 and H4 are retained, whereas ∼90% of core histones H2A and H2B are lost (Risley and Eckhardt 1981). This leaves *Xenopus* sperm with ∼10% of the amount of nucleosomal content of a spermatid. This level of histone retention in sperm is higher than that of mouse (∼1%) (Brykczynska et al. 2010), lower than that of zebrafish (∼100%) (Wu et al. 2011), and similar to that of human (∼10%) (Brykczynska et al. 2010). Nucleosome retention in vertebrates therefore seems to show a degree of variation among species. The epigenetic analysis of *Xenopus* sperm provided here extends the repertoire of characterized higher vertebrate sperm chromatin and identifies conserved chromatin features relevant to developmental programming. In that respect, we observed that the programming of sperm for embryonic gene expression entails a loss of H3K4me2/3 marking at H3K27me3 target genes during spermatid to sperm maturation (Fig. 6I). We showed that the set of genes programmed for embryonic expression during *Xenopus* sperm maturation had similar epigenetic features in *Xenopus* and human sperm (Figs. 4C, 5C). So, despite the existence of a hugely variable degree of histone retention in sperm among species, this points toward the existence of universal mechanisms preparing sperm for participation in the normal development of vertebrate embryos.

To functionally test the role of sperm epigenetic marks on embryonic gene expression, one would ideally like to erase these marks from the sperm nucleus immediately prior to the generation of embryos. Chromatin of mature sperm is highly condensed and inaccessible, making enzymatic treatments to alter the epigenetic marks inefficient. Alternative strategies have been developed to use such enzymes either during the process of spermiogenesis, prior to full maturation of sperm (Siklenka et al. 2015), or at fertilization when the sperm chromatin becomes accessible again (used in this study). In mouse, the former strategy has been used to overexpress the H3K4/H3K9 demethylase KDM1A in germ cells. Embryos generated with sperm from animals overexpressing KDM1A exhibited developmental defects which were transmitted for several generations in the absence of exogenous KDM1A. This analysis demonstrated the existence of epigenetic instruction delivered by sperm to the embryos and transmitted through several generations. However, the overexpression of KDM1A very early in the process of sperm differentiation leads to numerous abnormalities in sperm—for example, the presence of additional mRNAs (Siklenka et al. 2015). These abnormalities are indirect effects of the overexpression of KDM1A early in the process of sperm differentiation. For that reason, it has been difficult to link the difference in gene expression and associated developmental defects to particular epigenetic changes in sperm. The approach we describe here complements and extends previous analyses. First, by comparing the epigenetic profiles of sperm and spermatids to differential gene expression in sperm- and spermatid-derived embryos, we identified H3K4me2/3 and H3K27me3 as candidate marks responsible for the programming of genes. We then tested this hypothesis by demethylating the chromatin using KDM6B (H3K27 demethylase) or KDM5B (H3K4 demethylase) in embryos generated with sperm or spermatids. Importantly, in our experimental setup, both sperm and spermatids used had been through a normal differentiation process. Removal of H3K4me2/3 at fertilization affects different sets of genes in sperm- and spermatid-derived embryos. Genes affected in sperm-derived embryos are enriched for H3K27me3, whereas genes affected in spermatid-derived embryos are enriched for H3K4me2/3. This indicates the importance of H3K4me2/3 dynamics at the transition from spermatid to sperm for patterning of the future embryonic gene expression. One hypothesis to explain the sensitivity of sperm H3K27me3-marked genes to H3K4me2/3 removal would be that these genes acquire H3K4 methylation following fertilization. Our analysis also suggests a conserved role for these marks in *Xenopus* and mouse (Siklenka et al. 2015). Additionally, we also demonstrated that removal of H3K27me3 at fertilization affects the embryonic expression of genes that are marked by H3K27me3 in sperm/spermatids. Recent reports probing histone modifications distribution in mouse and human sperm suggested that these epigenetic marks occurred mostly on repetitive regions of the genome rather than genes (Carone et al. 2014; Samans et al. 2014). These observations put into question the possibility that such marks would influence gene expression in embryos. By providing a functional test of the need for histone modifications for embryonic gene expression, our analysis, together with that of Siklenka et al., clearly shows that, regardless of their genomic location, sperm-delivered modified histones are important regulators of expression in future embryos (Siklenka et al. 2015).

Further investigations into the nature of sperm programming, especially the requirement of other epigenetic marks and their cross-talk in the patterning of embryonic expression, will provide a better understanding of the transgenerational inheritance of epigenetic traits via gametes and could shed light on the mechanisms underlying male infertility and other diseases in humans.

## Methods

All the experiments involving the use of animals were conducted according to the regulatory standards of the funding bodies.

### Separation of sperm and spermatids

For each round of sperm and spermatid purification, testes from six adult *Xenopus laevis* males were isolated and manually cleaned from blood vessels and fat bodies in 1 × MMR (100 mM NaCl, 2 mM KCl, 2 mM CaCl_2_, 1 mM MgCl_2_, 5 mM HEPES, pH 7.4) using forceps and paper tissues. It is crucial to clean the testes well from any nontesticular tissues, as otherwise the cells released from the tissues may negatively affect the final purity of isolated cells. Subsequently, testes were torn into small pieces with forceps and homogenized with 2–3 strokes of a Dounce homogenizer (tissue from one testis at a time). The cell suspension was then filtered to remove tissue debris and cell clumps (CellTrics, cat. 04-0042-2317) and spun down at 800 rcf, 4°C, for 20 min. Supernatant was discarded and the cell pellet was resuspended in 12 mL of 1 × MMR. If any red blood cells were visible at the bottom of the pellet (a result of incomplete removal of blood vessels), only the uncontaminated part of the pellet was recovered, taking extreme care not to disturb the red blood cells. Subsequently, step gradients of iodixanol (Optiprep; Sigma, D1556; 60% iodixanol in water) in 1×MMR final were manually prepared in prechilled 14 mL ultraclear centrifuge tubes (Beckman Coulter, #344060) in the following order from the bottom to the top of the tube: 4 mL of 30% iodixanol, 1 mL of 20% iodixanol, 5 mL of 12% iodixanol (all in 1 × MMR), and 2 mL of cell suspension in 1 × MMR on top. Gradients were spun down in a prechilled SW40Ti rotor at 7500 rpm (10,000*g*), 4°C, for 15 min, deceleration without brake (Beckman Coulter Ultracentrifuge, Optima L-100XP). The top interface fraction (between 1 × MMR and 12% iodixanol), containing spermatids, and the pelleted fraction, containing mature sperm, were collected. Collected fractions were diluted six times with 1 × MMR and collected by spinning first at 805 rcf, 4°C, for 20 min and respinning at 3220 rcf, 4°C, for 20 min to pellet remaining cells. Pelleted cells were subjected to nuclei preparation (see below).

### Sperm and spermatid nuclei preparation, intra-cytoplasmic sperm injections to nonenucleated and to enucleated eggs and embryo culture

Sperm and spermatid nuclei were permeabilized as described before (Smith et al. 2006) and stored at −80°C. Injections were performed using a Drummond Nanoject microinjector (NanojectII Auto Nanolitre Injector, Biohit, 3-00-206A) and glass capillaries (Biohit, 3-00-203-G/XL) pulled using a Flaming-Brown micropipette puller (settings: heat 700, pull 100, velocity 100, time 10). The cell suspension was sucked into the injection needle filled with mineral oil. Cells were injected in sperm dilution buffer (SDB) (Smith et al. 2006), and cell concentration was adjusted by doing mock injections on a microscope slide to deliver one cell per 4.6 nL injection. The eggs were placed in batches of 20–25 on a blotting paper. If they were to be enucleated, they were placed with the animal pole facing upward, whereas if they were not subjected to enucleation, they were placed on a side (with the marginal zone upward). For enucleation, eggs were treated for 30 sec with a UV mineralite lamp (Gurdon 1960) (this step was omitted for nonenucleated eggs). Jelly was removed by a 5-sec Hanovia lamp treatment. The eggs were immediately injected with sperm or spermatid solution and moved to 1 × MBS (Gurdon 1976) supplemented with 0.2% bovine serum albumin (BSA). The cell suspension in the needle was replaced every 20–25 batches of eggs injected. At the four-cell stage, embryos were sorted (all the noncleaved embryos or those with irregular cleavage furrows were discarded) and the culture media replaced with 0.1 × MBS, 0.2% BSA. Embryos were cultured in 0.1 × MBS, 0.2% BSA (changed daily) in a 16°C–18°C incubator. Assessment of developmental stages was performed according to Nieuwkoop and Faber (Nieuwkoop and Faber 1994). Using this table, matching gastrula embryos from the various experimental groups were collected at stage 10^1/2^–11 and processed for gene expression analysis (see Supplemental Data procedures for details).

### Interphase egg extract preparation

Eggs were collected in 1 × MMR, de-jellied with 0.2 × MBS, 2% cysteine (pH 7.8–7.9) (Sigma, #W326305) and washed with 0.2 × MMR. Subsequently, eggs were activated for 3 min at room temperature (RT) with 0.2 × MMR supplemented with 0.2 µg/mL calcium ionophore (Sigma, #C7522). Eggs were rinsed with 0.2 × MMR, and subsequently all abnormal or not activated eggs were removed. Eggs were washed with 50 mL of ice-cold extraction buffer (EB) (5 mM KCl, 0.5 mM MgCl_2_, 0.2 mM DTT, 5 mM HEPES, pH 7.5) supplemented with protease inhibitors (PI) (Roche, #11873580001), transferred into centrifugation tubes (Thinwall, Ultra-Clear, 5 mL, 13 × 51-mm tubes, Beckman, #344057), supplemented with 1 mL of EB buffer with PI and 100 µg/mL of cytochalasin B (Sigma, #C2743), and placed on ice for 10 min. Subsequently, eggs were spun briefly at 350*g* for 1 min at 4°C (SW55Ti rotor, Beckman Coulter Ultracentrifuge, Optima L-100XP), and excess buffer was discarded. Eggs were then spun at 18,000*g* for 10 min at 1°C, the extract was collected with a needle, transferred to a fresh, prechilled tube, supplemented with PI and 10 µg/mL of cytochalasin B, and respun using the same conditions. Extract was collected with a needle and used fresh for the replication assay (see below).

### Replication in egg extracts and sample preparation for analysis of DNA fibers

Replication on single DNA fibers was performed as described before (Gaggioli et al. 2013) with slight modifications. Freshly prepared egg extracts were supplemented with 20× energy regeneration mix: 2 mg/mL creatine kinase (Roche, #10127566001), 150 mM creatine phosphate (Roche, #10621714001), 20 mM ATP (Roche, #10519979001), 2 mM EGTA, 20 mM MgCl_2_, and with 20 µM biotin-16-dUTP (Roche, #11093070910). Permeabilized cells were added to a final concentration of 200 nuclei/µL of extract and incubated at RT for 2 h (tapping every 10 min). The reaction was stopped by adding 10 volumes of ice-cold 1 × PBS (phosphate buffer saline: 137 mM NaCl, 2.7 mM KCl, 10 mM Na_2_HPO_4_ × 2H_2_O, 2 mM KH_2_PO_4_), and cells were spun down at 1000*g*, 4°C, for 7 min. Cells were resuspended in 50 µL of 1 × PBS and mixed immediately with 50 µL of melted (at 65°C) 2% low melting point agarose (Invitrogen, #16520050) in 1 × PBS. After solidification, the agarose plug was incubated overnight (O/N) at 50°C with 1 mL 0.5M EDTA, pH 8, 100 µL 10% sarkosyl (Sigma, #L5125), 1 mg/mL Proteinase K (New England Biolabs, #P8102S), followed by three washes in TE pH 6.5. Subsequently, the plug was incubated twice in TE supplemented with 0.1 mM PMSF (Sigma, #93482) for 30 min at 50°C and washed four times with 1 mL of 50 mM MES (Sigma, #69889), pH 6.35, 1 mM EDTA (1 h at RT each wash). Then, the solution was removed; the plug was melted in 400 µL of MES pH 6.35, 1 mM EDTA at 68°C for 20 min, and the agarose was digested with 2 units of β-agarase (New England Biolabs, #M0392S) O/N at 42°C.

### Analysis of replication on single DNA fibers

Silanized coverslips were prepared as described before (Labit et al. 2008). Thirty microliters of replicated DNA solution was pipetted onto a silanized coverslip, covered with a nonsilanized coverslip, and incubated for 5 min at RT. Subsequently, the top coverslip was slid away to stretch DNA fibers and the silanized coverslip with stretched fibers was fixed in a 3:1 solution of methanol:g lacial acetic acid for 10 min, RT. The fibers were then denatured with 2.5 M HCl (1 h, RT) and dehydrated by washes in 70% ethanol, 90% ethanol, and 100% ethanol (1 min for each wash). Subsequently, the coverslip was dried, washed three times in PBS, 0.1% Tween (Sigma, #P5927) (5 min for each wash) and blocked in 3% BSA in PBS (1 h, RT). All antibodies were diluted in PBS, 3% BSA, 0.1% Tween. Total DNA was detected simultaneously with replicated DNA with primary antibodies: anti-DNA antibody (Millipore, #MAB3034) 1:300 dilution, and streptavidin-Alexa 594 antibody 1:50 to detect biotin (Invitrogen, #S-11227) for 30 min at 37°C. Primary antibodies were washed away with PBS, 0.1% Tween (four washes) and detected with secondary antibodies diluted 1:50: chicken anti-mouse Alexa 488 (Invitrogen, #A-21200) and biotinylated antibody anti-streptavidin (Vector Labs, #BA-0500) for 30 min, 37°C. After four washes in PBS, 0.1% Tween, a tertiary detection was performed with antibodies diluted 1:50: goat anti-chicken Alexa 488 (Invitrogen, #A-11039) and streptavidin-Alexa 594 for 30 min, 37°C. The coverslip was washed three times with PBS, 0.1% Tween, three times in PBS, mounted on a microscope slide with a mounting medium (50% glycerol in PBS), and sealed with nail polish. Images were acquired with a Zeiss 510 META confocal LSM microscope. Image analysis was performed in ImageJ; the amount of replicated DNA and total DNA was measured individually on single DNA fibers.

### RNA extraction and preparation of cDNA library for sequencing

Spermatid (1 million) or a pool of five stage 10.5–11.5 embryos were collected and frozen at −80°C. RNA extractions were performed using a Qiagen RNeasy Mini Kit according to the manufacturer's protocol. RNA was eluted in 50 µL of DEPC H_2_O and used to generate cDNA sequencing libraries using an Illumina TruSeq Kit (#RS-122-2001), according to the manufacturer's protocol.

### mRNA injection to one-cell embryos

Mouse KDM6B (aa1025–1642) or KDM5B (aa1–770) were cloned using the p-Entry cloning system (Invitrogen, #K2400-20 and 11791-020) into a pCS2+ plasmid with a C-terminal HA-tag and NLS-tag. mRNA was synthesized in vitro using a MEGAscript SP6 Kit (Ambion, AM1330M) following the manufacturer's instructions. Eggs were in vitro–fertilized and de-jellied using a 2% cysteine solution in 0.1 × MMR. Injections into one-cell stage embryos were performed in injection solution (Smith et al. 2006) using a Drummond Nanoject microinjector, delivering 9.2 ng of mRNA per injection (mRNA at 1 mg/mL in DEPC H_2_O). Embryos were cultured at 18°C and collected for Western blot analysis at stage 21 (Nieuwkoop and Faber 1994). Western blot analyses were performed on 12% polyacrylamide gels using antibodies against H3K27me3 (Cell Signalling, #9733), H3K9me2/3 (Cell Signalling, #5327), H3K4me2/3 (Abcam, #8580), H4 (Abcam, #31830), and against H3 (Abcam, #18521)

### Preparation of ChIP-seq samples

Sperm and spermatids were separated as described above. Chromatin fractionation and chromatin immunoprecipitation (ChIP) were performed as described before (Erkek et al. 2013; Hisano et al. 2013) with slight modifications. Pretreatment of sperm cells with DTT was omitted, and chromatin was digested with 2.5 U of MNase/1 million of cells (Roche, #12533700) for 30 min at 37°C. The following antibodies against histone marks were used in the study: anti-H3K4me2 (Millipore, #07-030), anti-H3K4me3 (Abcam, #ab8580), anti-H3K4me3 (Millipore, #CS200580), anti-H3K27me3 (Millipore, #07-449), anti-H3K27me3 (kind gift from Dr. Thomas Jenuwein), and anti-H3K9me3 (Abcam, #ab8898). Before ChIP, primary antibodies were bound to magnetic beads conjugated with secondary antibody (Invitrogen, #11204D) according to the manufacturer's protocol, and all wash steps in the protocol were carried out with a magnet, instead of centrifugation. Bound DNA was isolated, separated by electrophoresis, and mononucleosomal bands from sperm and spermatids were excised and subjected to library preparation with a TruSeq DNA Kit (Illumina, #FC-121-2001). For the generation of the input sample, 5%–10% of the MNase-digested chromatin was collected, and the same purification scheme was followed as with the immunoprecipitated chromatin prior to library preparation with a TruSeq DNA Kit (Illumina, #FC-121-2001).

### Preparation of MBD-seq samples

Sperm and spermatid chromatin were separated as described above, and 200 ng of digested genomic DNA were used to purify methylated DNA using the Methyl Collector TM Ultra Kit (Active Motif, #55005). The purification was carried out according to the manufacturer's instructions, using the low salt buffer to wash the bead-methyl DNA complexes. The purified methylated DNA and the input DNA were then subjected to library preparation with the TruSeq DNA Kit (Illumina, #FC-121-2001).

### Sequencing data analysis

Details of the sequencing data analysis methods used in this study are described in the Supplemental Material.

## Data access

All ChIP-seq, RNA-seq, MBD-seq, and MNase-seq data sets from this study have been submitted to the NCBI Gene Expression Omnibus (GEO; http://www.ncbi.nlm.nih.gov/geo/) under accession number GSE75164.
